# Understanding co-production of injury research in Australian Aboriginal and Torres Strait Islander communities: a comprehensive scoping review

**DOI:** 10.1186/s40621-024-00556-8

**Published:** 2025-01-07

**Authors:** Genevieve Westacott, Victoria McCreanor, Susanna Cramb, Silvia Manzanero, Kim Vuong, Michelle Allen, Shannon Dias, Geoffrey Binge, Arpita Das

**Affiliations:** 1 Jamieson Trauma Institute, Royal Brisbane and Women’s Hospital, Metro North Health, L13, Block 7, Herston, Brisbane, QLD 4029 Australia; 2 Hunter Medical Research Institute, Newcastle, Australia; 3 School of Public Health and Health Services, Queensland University of Technology, Brisbane, Australia; 4 School of Clinical Sciences, Queensland University of Technology, Brisbane, QLD Australia; 5 Australian Centre for Health Services Innovation, Centre for Healthcare Transformation, Faculty of Health, Queensland University of Technology, Brisbane, Australia; 6 Forensic Mental Health Group and Military and Veterans’ Mental Health Collaborative, Brisbane, Australia; 7 Queensland Centre for Mental Health Research, West Moreton Health, Brisbane, Australia; 8 Indigenous Strategic Development Department, Royal Brisbane and Women’s Hospital, Metro North Health, Brisbane, Australia

**Keywords:** Injury, Aboriginal, Torres Strait Islander, First Nations, Indigenous, Co-production, Australia

## Abstract

**Background:**

Injury causes significant burden on Australian Aboriginal and Torres Strait Islander communities. However, a considerable portion of the research conducted in this area has been carried out by Western researchers. It has been acknowledged that historical research methodologies and discourses around Aboriginal and Torres Strait Islander research may not be suitable or beneficial. Co-production methodologies offer opportunities for research to be developed collaboratively ensuring meaningfulness of results and appropriate protection for participants. A scoping review was undertaken to investigate the use of co-production methodologies in research within the unintentional injuries space for Australian Aboriginal and Torres Strait Islander communities over time.

**Main body:**

A systematic search was conducted using PubMed, ProQuest, Embase and Indigenous Health Infonet databases. Study characteristics, remoteness, injury topic, co-production methods and elements were extracted from eligible studies. The search revealed 4175 papers, from which 39 studies were included in this scoping review. It was found that 69% of studies were fully co-produced with community. Studies predominately focused on general injury, falls prevention or brain injury rehabilitation. The most heavily utilised co-production strategy was the inclusion of Aboriginal and Torres Strait Islander researchers into the writing and research team. This helped the collection of culturally safe data and appropriate interpretation of results.

**Conclusion:**

There is growing diversity among co-production methodologies, better enabling meaningful engagement between community and research. This co-production helps decolonise the research process to privilege Aboriginal voices, however, more work is needed to appropriately capture Indigenous perspectives.

**Supplementary Information:**

The online version contains supplementary material available at 10.1186/s40621-024-00556-8.

## Background

Indigenous peoples and communities throughout the world are some of the most heavily researched populations [1]. However, most research to date has tended to focus on and utilise Western methodologies, meaning that Indigenous peoples were the research subjects and were actively excluded from the research designing, producing and decision-making processes. Additionally, much of the research has been conducted to solely benefit the researchers and Western Knowledge Systems, providing little practical benefit to communities. Findings produced in this way are misrepresentative of issues due to inherent bias within Western Methodologies such as, racism and failure to understand or value different cultural views enforcing ‘solutions’ through a Western lens [2, 3].

This is true for research about Aboriginal and Torres Strait Islander peoples in Australia. Historical events and continuing racist practices have made research a dirty word for many. Due to ineffective policies and forced Western agendas, research has often perpetuated inequities and poor health outcomes for Aboriginal and Torres Strait Islander peoples [4, 5]. Since Australia’s colonisation in 1788, Aboriginal and Torres Strait Islander communities have been studied and researched to the point of exploitation across various fields (e.g., anthropology, health) [1, 6]. In recent years, there has been a shift towards self-determination and enabling appropriate sovereignty over Aboriginal and Torres Strait Islander governance and leadership [7]. Alternative, modified, and enhanced research methodologies are becoming more common in literature as they change the current narrative, allow for truth-telling in research and ensure new investigation is culturally appropriate and aware [2, 5]. Approaches such as co-design, community participatory research and community-led research allows for more meaningful investigation outcomes and outputs as community have a formalised opportunity to express their priorities and needs.

Injury is a leading cause of death and disability in Australia, contributing 8.1% of the overall burden of disease [8]. Between 2021 and 2022, there were over 31,000 hospitalisations and 500 deaths recorded within Aboriginal and Torres Strait Islander communities due to traumatic injury, putting strain on families, communities, and services to help manage care. Understanding the breadth and scope of research about injuries to Aboriginal and Torres Strait Islander people in Australia, and the extent to which community members have been approached, engaged, or included in the research production, will help identify potential gaps in the injury literature [9].

## Co-production in research

The term ‘co-production’ is a description of methodology which utilises collaboration, partnership and engagement between researchers and study participants to achieve research goals [10]. It is underpinned by key principles such as empowerment and shared power, capacity building and reciprocity, and co-creation. Co-production, by definition, includes co-design methods and goes further to include the end or production of research [11]. Co-production strongly aligns with participatory research as common themes include equal power and decision making, and community leadership [10].

Within Aboriginal and Torres Strait Islander research, co-production has become an essential methodology to conduct research, as it embeds the voices of the community in the research [7]. Co-production helps to stop the exclusion of Aboriginal and Torres Strait Islander peoples from research (except as a research subject), better enabling self-determination for communities [12, 13].

Elements of co-production can occur at any point of the research process and can be incorporated into the methodology. Ideally, co-production elements and Indigenous Knowledge systems will be integrated into all research phases including the design, analysis, interpretation, and dissemination; but, as yet are not standard practice across all Aboriginal and Torres Strait Islander research studies.

However, co-production methods look different between mainstream Australia and Aboriginal and Torres Strait Islander specific research due to the history of exclusion. This has resulted in many forms of co-production methods which vary from minimal engagement in the research process to fully co-produced/collaborative research studies [10].

The primary aim of this scoping review is to understand the recent published information about unintentional injuries to Aboriginal and Torres Strait Islander peoples and the extent communities were involved in the research process. Objectively, trends will be analysed over time to observe changes to co-production inclusion in research methodology.

## Main text

### Methods

This scoping review followed the Preferred Reporting Items for Systematic reviews and Meta-Analyses extension for Scoping Reviews (PRISMA-ScR) framework to ensure rigour in the undertaken steps [14]. The preliminary protocol was registered at the Open Science Framework [15].

### Eligibility criteria

Publications were included if they related to frequency, cause, rehabilitation, or prevention of unintentional injuries, and pertained to Aboriginal and Torres Strait Islander communities or peoples as either the cohort or a sub-group. Peer-reviewed studies of any methodology were included if they were written in English and published from 2010 onwards to ensure relevancy of information. Studies were excluded if they examined intentional injury, such as self-harm or assault, or did not include any co-production elements. For more details, see Table 1.

**Table 1:** Inclusion and exclusion criteria for paper screening and assessment

	Inclusion	Exclusion
Population	Australian Aboriginal and Torres Strait Islander peoples and communities, including as a subset	Non-Indigenous communities
Phenomenon of interest	Unintentional injury information and research, including topics of prevention	Intentional injury information, non-injury research or injury resulting in death. Additionally, mental health or psychological trauma
Comparison	Mainstream Australian/non-Indigenous communities	Non-Australian communities
Evaluation	Information about co-production with Aboriginal and Torres Strait Islander peoples and communities	No information about co-production with Aboriginal and Torres Strait Islander peoples and communities
Timeframe	Published from 1 January 2010	Published before 2010
Language	Published in English	Not available in English
Article type	Peer-reviewed publications; qualitative methodology, quantitative methodology, mixed-methods methodology	Conference abstracts, letters, newspaper articles, protocols, or reviews

Source: developed by authors

### Search strategy

PubMed, ProQuest, Embase and Indigenous Health Infonet databases were searched for relevant articles published between January 1, 2010, and June 12, 2024 using the developed search strategy in Table 2. During the development of the search strategy other databases and libraries were searched including CINAHL, Web of Science, Informit, Wiley Online, and Sage, but they did not yield any relevant results.

**Table 2:** Search strategy used in this scoping review

aborig* OR ‘torres strait’ OR ‘first nation*’ OR ‘first people*’ or ‘indigen*’
AND
injur* OR traum* OR ‘multi traum*’ OR ‘multi-traum*’ OR multitraum* OR accident OR sport OR ‘toxic substance’ OR damage OR harm OR hurt
AND
“co-design’ OR “co design” OR codesign OR “co-production” OR “co production” OR coproduction OR collabor* OR participatory OR co-creat* OR “community led” OR community-led OR “indigenous method” OR “indigenist method” OR shared
NOT
Canada OR Canadian OR Hawaii OR Hawaiian OR America OR American

Source: developed by authors

Searching the PubMed and Embase databases meant that both MeSH and EMTREE terms could be utilised to enhance the capability and reach of the search strategy.

### Screening process

The online tool Covidence [16] was used to undertake the screening process. One reviewer completed the title and abstract screen against the eligibility criteria (Table 1). Five percent of the studies excluded during the title and abstract screen were reviewed by a second reviewer, to ensure there were no eligible studies excluded. A full text screening was completed by two independent reviewers. Conflicts and disagreements were resolved through mutual agreement amongst the review team.

### Data extraction and assessment

Data from eligible studies were extracted to a Microsoft Excel spreadsheet. Key information about each selected article was extracted including first author, year of publication, study population and demographics and remoteness of target region. Injury information extracted included: injury topic, the part of the patient journey focused on and the type of data utilised (i.e., newly collected data or accessing of pre-existing data).

To enable better analysis, age ranges were grouped as:

- Babies and toddlers (0–4 years of age)
- Children (5–14 years of age)
- Adolescents (15–17 years of age)
- Young Adults (18–29 years of age)
- Adults (30–69 years of age)
- Elderly (70+ years of age)

Lastly, the co-production methods and elements used in the research was extracted. Table 3 contains a list of the potential elements utilised by research articles. The development of this list of co-production elements was led by our Aboriginal author, with influence from Butler et al.’s [10] body of work. Once data extraction commenced, any perceived additional elements of co-production outside of this list were discussed by the team and included.

**Table 3:** Potential co-production elements grouped by the phase of research they are likely to be completed

Research phase	Co-production element
Conceptualisation	• Advisory group engagement or community consultation
Design and planning	• Indigenous Methods or decolonising Methodologies (excludes participatory research)
Empirical	• Aboriginal or Torres Strait Islander research clinician or research assistant • Use of Aboriginal or Torres Strait Islander research tools (such as culturally safe survey)
Analysis and dissemination	• Aboriginal or Torres Strait Islander authorship • Aboriginal or Torres Strait Islander organisation affiliation • Use of Aboriginal or Torres Strait Islander analytic tools (such as Harfield et al.) • Feedback or editing from Aboriginal or Torres Strait Islander persons
Across all phases	• Aboriginal or Torres Strait Islander leadership/community-led research or participatory research • Aboriginal or Torres Strait Islander persons on the research team • Continuous engagement with Aboriginal and Torres Strait Islander advisory group or committee, or community consultation

Source: developed by authors

All components and sections of the paper were investigated to extract any perceived elements of co-production, including the searching of authors online, if it was not stated within the study, to find out if they identified as an Aboriginal or Torres Strait Islander people. If the information was not clearly explained or not easily found through an internet search, it was assumed the co-production element was not present. A level or amount of co-production was quantified for each study based on the information provided in each study. Studies were considered fully co-produced if it was stated within the study that co-production elements occurred across all phases (e.g., there was clear consultation with an advisory group across the whole research process), or an element was used in each phase of the research. The use of Indigenous or decolonising methods by itself was not considered to be a full co-production element as these methods can be utilised by any research team.

Information will be grouped to enhance analysis of results as set out in Table 3.

### Critical appraisal of selected studies

This research is a scoping review, so a critical appraisal of the selected studies was not undertaken. However, the captured and portrayed Aboriginal and Torres Strait islander perspective was analysed and quantified for each study using Harfield et al. [17]. Questions that were answered yes were given 1 point, partial 0.5, no or unclear 0. This score was then averaged for level of Aboriginal and Torres Strait islander perspective captured; none—low (0–33%), medium (34–66%), or high (67–100%). Answers of unclear were tallied to identify amount of uncertainty.

## Results

**Fig. 1:**
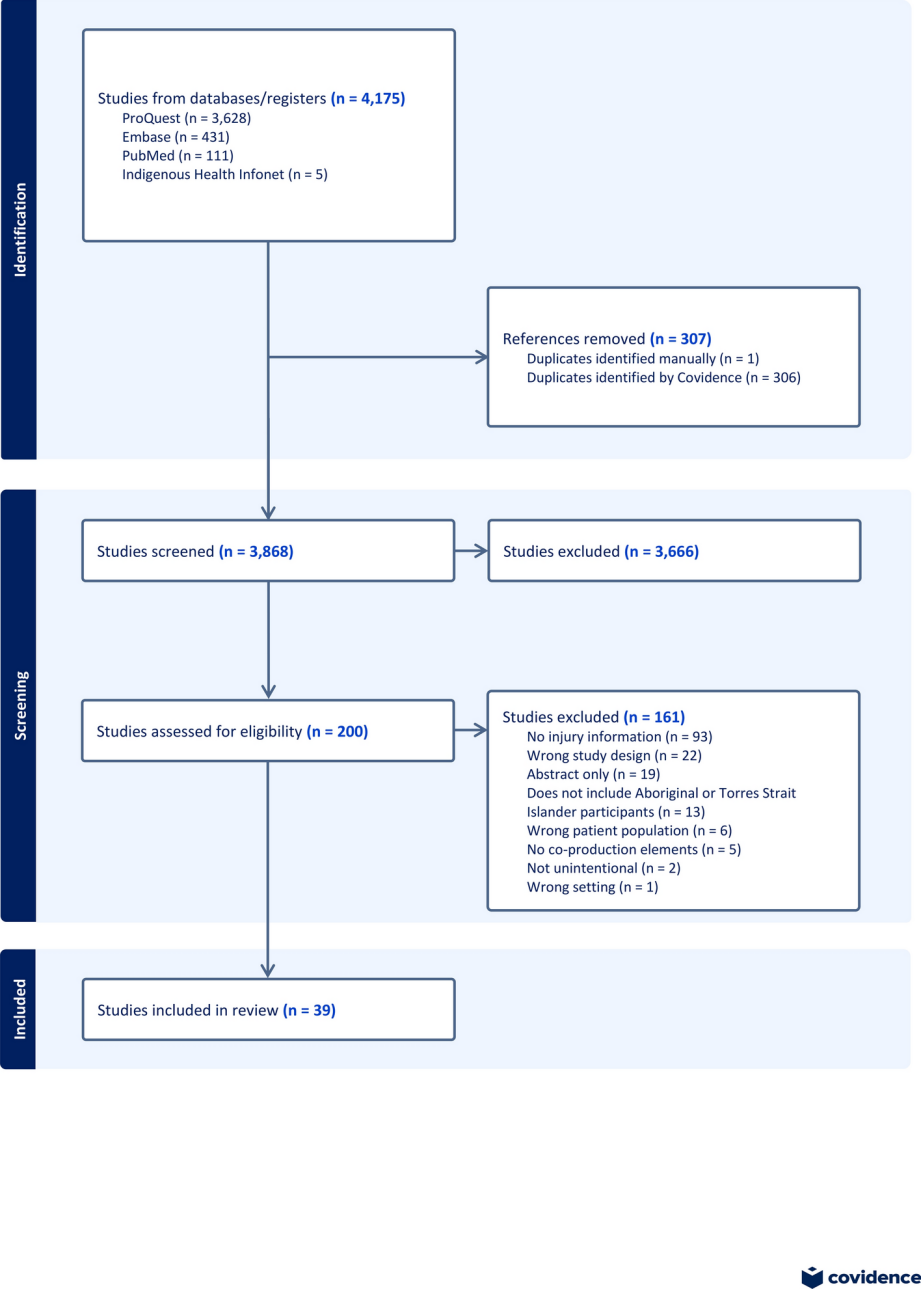
PRISMA-ScR flowchart of paper screening process. Source: Formatting developed using [14], content developed by authors

The PRISMA flowchart in Fig. 1 shows the review process. The searches resulted in a total of 4175 records and after duplicates were removed, 3868 papers remained to be screened. After screening titles, abstracts, and keywords for eligibility, 200 studies required full text screening. Of those, 39 were included in the review.

### Study characteristics

For all study characteristics and injury information please refer to Table 4.

**Table 4:** Study characteristics and injury information of the selected studies in this review

Article #	First author	Year	Study type	Age group	Region/s	Remoteness	Target population	Injury topic	Patient journey	Data type
1	Angell [18]	2018	Mixed methods	Adults Elderly	NSW	Metro	Full cohort	Falls	Prevention Community-based	New data *Survey or questionnaire*
2	Armstrong [19]	2021	Randomised Control Trial	Young Adults Adults Elderly	WA	Metro Regional	Full cohort	Brain Injury	Rehabilitation Community-based	New data *Survey or questionnaire*
3	Bohanna [20]	2019	Qualitative longitudinal	Adults	QLD NT	Unspecified	Full cohort	Brain Injury	Rehabilitation Community-based	New data *Survey or questionnaire*
4	Cheok [21]	2023	Retrospective cohort study	All ages	NT	Rural	Full cohort	General injury	Discharge element	Pre-existing data
5	Clapham [22]	2017	Qualitative program evaluation	Babies and toddlers	NSW	Metro	Full cohort	General injury	Community-based	New data Survey or questionnaire
6	Cochrane [23]	2024	Qualitative	Young Adults Adults Elderly	QLD	Regional	Full cohort	Brain Injury	Rehabilitation Hospital admission	New data *Survey or questionnaire*
7	Coombes [24]	2020	Qualitative	All ages	SA NT QLD NSW	Multiple locations	Full cohort	Burns	Entire patient journey	New data *Survey or questionnaire*
8	Cotter [25]	2012	Cross sectional study	All ages	National	Multiple locations	Subset of cohort (1% Aboriginal and Torres Strait Islander peoples)	Fracture	Other	Pre-existing data
9	Dossetor [26]	2021	Population-based study	Babies and toddlers Children	WA	Remote	Subset of cohort (95% Aboriginal and Torres Strait Islander peoples)	General injury	Prevention Emergency presentation	Pre-existing data
10	Edmonston [27]	2020	Qualitative	Adolescents Young adults Adults Elderly	QLD	Rural Remote	Subset of cohort (35% Aboriginal and Torres Strait Islander peoples)	Road Trauma	Other	New data *Survey or questionnaire*
11	Esgin [28]	2023	Participatory action research	Young adults Adults	WA	Metro	Full cohort	General injury	Community-based	New data *Survey or questionnaire*
12	Falster [29]	2013	Retrospective cross sectional	All ages	NSW	Multiple locations	Subset of cohort (2.7% Aboriginal and Torres Strait Islander peoples)	Road Trauma	Hospital admission Discharge element	Pre-existing data
13	Fraser [30]	2021	Qualitative cross sectional	All ages	National	Multiple locations	Full cohort	Burns	Hospital admission	New data *Survey or questionnaire*
14	Gauld [31]	2011	Participatory action research	All ages	QLD	Remote	Full cohort	Brain Injury	Prevention Rehabilitation Community-based	New data
15	Hill [32]	2016	Cross sectional study	Adults Elderly	WA	Remote	Full cohort	Falls	Prevention Community-based	New data *Survey or questionnaire*
16	Katzenellenbogen [33]	2018	Cross sectional study	Adolescents Young adults Adults	WA	Multiple locations	Subset of cohort (13.8% Aboriginal and Torres Strait Islander peoples)	Brain Injury	Hospital admission Rehabilitation	Pre-existing data
17	Keel [34]	2017	Cross sectional study	Adults Elderly	National	Multiple locations	Subset of cohort (36% Aboriginal and Torres Strait Islander peoples)	Ocular trauma	Community-based	Both
18	Lee [35]	2018	Retrospective cohort study	Babies and toddlers	NSW	Multiple locations	Subset of cohort (3.7% Aboriginal and Torres Strait Islander peoples)	Poisonings	Admitted	Pre-existing data
19	LoGiudice [36]	2012	Cross sectional study	Adults Elderly	WA	Remote	Full cohort	Falls	Community-based	New data *Survey or questionnaire*
20	Lukaszyk [37]	2016	Qualitative	Adults Elderly	NSW	Multiple locations	Full cohort	Falls	Prevention Community-based	New data *Survey or questionnaire*
21	Lukaszyk [38]	2018	Mixed methods	Adults Elderly	NSW	Multiple locations	Full cohort	Falls	Prevention Community-based	New data *Survey or questionnaire*
22	Lukaszyk [39]	2017	Qualitative	Adults Elderly	NSW	Multiple locations	Full cohort	Falls	Prevention Rehabilitation Community-based	New data *Survey or questionnaire*
23	Lukaszyk [40]	2017	Retrospective population-based study	Adults Elderly	NSW	Multiple locations	Subset of cohort (0.85% Aboriginal and Torres Strait Islander peoples)	Falls	Hospital admission	Pre-existing data
24	Lukaszyk [41]	2018	Retrospective cohort study	Adults Elderly	NSW	Metro Regional	Full cohort	Falls	Prevention Community-based	New data *Survey or questionnaire*
25	McAuley [42]	2016	Prospective population-based study	Babies and Toddlers	WA	Multiple locations	Subset of cohort (5.4% Aboriginal and Torres Strait Islander peoples)	General injury	Emergency presentation Hospital admission	New data Hospital ICD-based
26	Moller [43]	2017	Retrospective cohort	Babies and toddlers Children	NSW	Multiple locations	Subset of cohort (3.2% Aboriginal and Torres Strait Islander peoples)	General injury	Emergency Admitted	Pre-existing data
27	Moller [44]	2016	Retrospective cohort	Babies and toddlers Children	NSW	Multiple locations	Subset of cohort (3.2% Aboriginal and Torres Strait Islander peoples)	General injury	Emergency Admitted	Pre-existing data
28	Moller [45]	2017	Population-based study	Babies and toddlers Children	NSW	Multiple locations	Subset of cohort (3.1% Aboriginal and Torres Strait Islander peoples)	Burns	Emergency Admitted	Pre-existing data
29	Moller [46]	2019	Retrospective cohort	Babies and toddlers Children	NSW	Multiple locations	Subset of cohort (3.2% Aboriginal and Torres Strait Islander peoples)	General injury	Emergency Admitted	Pre-existing data
30	Phillips [47]	2022	Qualitative	Babies and Toddlers Children Adolescents	QLD	Region Rural Remote	Subset of cohort (25% Aboriginal and Torres Strait Islander peoples)	Burns	Rehabilitation Other outpatient care	New data *Survey or questionnaire*
31	Ryder [48]	2021	Retrospective cohort study	Babies and Toddlers Children	National	Multiple locations	Subset of cohort (10.4% Aboriginal and Torres Strait Islander peoples)	Burns	Prevention Hospital admission	Pre-existing data
32	Ryder [49]	2020	Retrospective cohort study	Babies and Toddlers Children	National	Multiple locations	Subset of cohort (10.4% Aboriginal and Torres Strait Islander peoples)	Burns	Hospital admission	Pre-existing data
33	Schultz [50]	2018	Qualitative	Young Adults Adults Elderly	WA NT	Remote	Subset of cohort (89% Aboriginal and Torres Strait Islander peoples)	General injury	Prevention Community-based	New data *Survey or questionnaire*
34	Shepherd [51]	2012	Cross sectional study	Babies and Toddlers Children Adolescents	WA	Multiple locations	Full cohort	General injury	Community-based	Both *Survey or questionnaire*
35	Smith [3]	2023	Case Study	Adults	NT	Unspecified	Full cohort	Brain Injury	Rehabilitation Other outpatient care	New data *Observation*
36	Thurber [52]	2018	Cross sectional study	Babies and Toddlers Children Adolescents	NSW	Metro	Full cohort	General injury	Prevention Community-based	Pre-existing data
37	Veli-Gold [53]	2020	Qualitative	Adolescents Young Adults Adults	QLD	Unspecified	Subset of cohort (48% Aboriginal and Torres Strait Islander peoples)	Brain Injury	Emergency presentation	Pre-existing data
38	Wallis [54]	2015	Retrospective population-based study	Babies and Toddlers Children Adolescents	QLD	Multiple locations	Subset of cohort (81% Aboriginal and Torres Strait Islander peoples)	Drowning	Prevention Entire patient journey	Pre-existing data
39	Williams [55]	2021	Qualitative	Babies and Toddlers Children	National	Multiple locations	Full cohort	Burns	Hospital admission	New data *Survey or questionnaire*

The two most frequently used methods in the reviewed studies were qualitative (33%) [20, 22–24, 27, 30, 37, 39, 47, 50, 53, 55] and cross-sectional design (27%) [25, 29, 30, 32–34, 36, 51, 52], with one qualitative cross sectional study [30]. Most studies preferred to collect new data (54%) [3, 18–20, 22–24, 27, 28, 30–32, 36–39, 41, 42, 47, 50, 55] rather than accessing pre-existing datasets (41%) [21, 25, 26, 29, 33, 35, 40, 43–46, 48, 49, 52–54], and two studies used a combination of both [34, 51].

Surveys and questionnaires were the most common data collection methods for new data, as they could be incorporated into interviews, face-to-face discussions and yarning circles (83%) [18–20, 22–24, 27, 28, 30, 32, 36–39, 41, 47, 50, 51, 55].

New South Wales (38%) [18, 22, 24, 29, 35, 37–41, 43–46, 52] and Western Australia (23%) [19, 26, 28, 32, 33, 36, 42, 50, 51] were the most studied areas, excluding six nation-wide studies [25, 30, 34, 48, 49, 55]. There were no studies that focused on regions within Victoria, Tasmania and the Australian Capital Territory outside of national studies (some of which also excluded some of these regions) [25].

While studies primarily investigated multiple regions with varying remoteness (54%) [24, 25, 29, 30, 33–35, 37–40, 42–46, 48, 49, 51, 54, 55], 21% of studies specified investigating rural and remote regions [21, 26, 27, 31, 32, 36, 47, 50]. There were two studies that collected and compared information between metropolitan and regional areas [19, 41].

The majority of studies were published between 2017 and 2018 (33%) [18, 22, 33–35, 38–41, 43, 45, 50, 52], however, there is a clear spike in publications between 2020 and 2021 (23%) [19, 24, 26, 27, 30, 48, 49, 53, 55].

Among 39 studies, 18 studies (51%) targeted exclusively Aboriginal and Torres Strait Islander populations [3, 18–24, 28, 30–32, 36–39, 41, 51, 52, 55], and 49% [19] targeted mainstream communities with Aboriginal and Torres Strait Islander populations as a subgroup [25–27, 29, 33–35, 40, 42–50, 53, 54]. In the latter studies, the average participant representation from Aboriginal and Torres Strait Islander communities was 28%, ranging from less than 1% to 95%. Studies that targeted specific regions were more likely to investigate Aboriginal and Torres Strait Islander peoples only.

Participant ages ranged from less than 12 months to over 90 years. Adults (46%) [3, 18–20, 23, 27, 28, 32–34, 36–41, 50, 53] and the elderly (33%) [18, 19, 23, 27, 32, 34, 36–41, 50] were the most commonly investigated age groups. Seven studies [22, 30, 37, 42, 47, 51, 55] that collected new data did not engage the study cohort directly. Five of these studies focused on babies, children or adolescents and gathered information from caregivers or clinicians [22, 42, 47, 51, 55]. One of the studies focused on older adults and falls, and primarily used information from clinicians [37].

### Injury topic

General or unspecified injury was most frequently investigated (28%), however, this tended to be a broader overview of injury as a whole [21, 22, 26, 28, 42–46, 50–52]. Falls were the main focus (21%) [18, 32, 36–41] of most studies, with brain injury (18%) [3, 19, 20, 23, 31, 33, 53] and burns (18%) [24, 30, 45, 47–49, 55] also receiving much interest.

Injury topics are evenly distributed across the time frame with falls studies peaking in 2018 (3) [18, 38, 41] and burns studies peaking in 2021 (3) [30, 48, 55]. Interestingly, road trauma [27, 29] and drowning [54] studies only had minimal to some co-production.

Injury prevention was included in 46% of studies [18, 22, 26, 31, 32, 35, 37–39, 41, 43–46, 48, 50, 52, 54], most often discussed when investigating general injuries [22, 26, 43, 44, 46, 50, 52] and falls [18, 32, 37–39, 41] as described in Table 5. Over 70% of the studies that included injury prevention were fully co-produced [18, 22, 26, 31, 32, 37–39, 41, 43, 48, 50, 52] and half involved the collection of new data [18, 22, 31, 32, 37–39, 41, 50]. Additionally, topics of prevention appear to be declining with 83% of studies published before 2019 [18, 22, 31, 32, 35, 37–39, 41, 43–46, 50, 52, 54].

**Table 5:** Number of studies by injury, level of co-production, and inclusion of injury prevention within study

Injury topic	Not injury prevention			Injury prevention			Total
	Full	Some	Minimal	Full	Some	Minimal	
General injury	3	1	0	5	2	0	11
Falls	0	2	0	6	0	0	8
Burns	4	1	0	1	1	0	7
Brain injury	5	0	1	1	0	0	7
Road trauma	0	1	1	0	0	0	2
Ocular trauma	1	0	0	0	0	0	1
Drowning	0	0	0	0	0	1	1
Poisonings	0	0	0	0	1	0	1
Fracture	1	0	0	0	0	0	1
Total	14	5	2	13	4	1	39

Source: developed by authors

Rehabilitation was included in 21% of studies [3, 19, 20, 23, 31, 33, 39, 47] and only for brain injury, falls and burns studies. Three-quarters of the rehabilitation-oriented articles are fully co-produced [19, 20, 23, 31, 33, 39] and 88% collected new data [3, 19, 20, 23, 31, 39, 47]. See Table 6 for further information.

**Table 6:** Number of studies by injury, level of co-production, and inclusion of injury rehabilitation within study

Injury topic	Not rehabilitation			Rehabilitation			Total
	Full	Some	Minimal	Full	Some	Minimal	
General injury	8	3	0	0	0	0	11
Falls	5	2	0	1	0	0	8
Burns	5	1	0	0	1	0	7
Brain injury	1	0	0	5	0	1	7
Road trauma	0	1	1	0	0	0	2
Ocular trauma	1	0	0	0	0	0	1
Drowning	0	0	1	0	0	0	1
Poisonings	0	1	0	0	0	0	1
Fracture	1	0	0	0	0	0	1
Total	21	8	2	6	1	1	39

Source: developed by authors

A little under half of the studies investigated information around community health or community-based services (41%) [18–20, 22, 28, 31, 32, 34, 36–39, 41, 50–52]. This aligns with the injury focus, as seven studies which target falls focused on community-based programs or services [18, 32, 36–39, 41] and five investigated general injury [22, 28, 50–52]. Studies focusing on community-based services were more likely to have fully co-produced research (88%) [18–20, 22, 28, 31, 32, 34, 37–39, 41, 50, 52], with no studies in the minimal co-production category. Additionally, there was one study which focused on hospitalisations post-car crash rather than a specific part of the patient journey [27] and another focused on age-related conditions rather than a point of care [25].

### Co-production methodologies

Please refer to Table 7 for the co-production methodology information.

**Table 7:** Co-production information from the selected studies in this review

Article #	First author	Conceptualisation	Design and planning	Empirical	Analytic and dissemination	Full co-production elements	Total level
1	Angell [18]		Indigenous/decolonising methods Indigenous specific ethics committee	Identified research clinician /research assistant	Authorship, feedback or editing	Identified team members	Full
2	Armstrong [19]		Indigenous specific ethics committee	Identified research clinician /research assistant	Authorship, feedback or editing Affiliation	Identified team members	Full
3	Bohanna [20]	Advisory group/consultation	Indigenous/decolonising methods	Indigenous developed research tools	Authorship, feedback or editing		Full
4	Cheok [21]			Identified research clinician /research assistant	Authorship, feedback or editing Affiliation	Identified team members	Full
5	Clapham [22]	Advisory group/consultation	Indigenous/decolonising methods Indigenous specific ethics committee	Identified research clinician /research assistant	Authorship, feedback or editing	Identified team members	Full
6	Cochrane [23]		Indigenous/decolonising methods Indigenous specific ethics committee	Identified research clinician /research assistant Indigenous developed research tools	Authorship, feedback or editing	Identified team members	Full
7	Coombes [24]		Indigenous/decolonising methods Indigenous specific ethics committee	Identified research clinician /research assistant	Authorship, feedback or editing	Identified team members	Full
8	Cotter [25]				Authorship, feedback or editing Affiliation	Identified team members	Full
9	Dossetor [26]		Indigenous specific ethics committee		Authorship, feedback or editing Affiliation	Identified team members	Full
10	Edmonston [27]	Advisory group/consultation	Indigenous/decolonising methods				Some
11	Esgin [28]		Indigenous/decolonising methods Indigenous specific ethics committee	Indigenous developed research tools	Authorship, feedback or editing Affiliation	Community led or participatory research Identified team members	Full
12	Falster [29]	Advisory group/consultation					Minimal
13	Fraser [30]		Indigenous/decolonising methods Indigenous specific ethics committee	Identified research clinician /research assistant	Authorship, feedback or editing Affiliation	Identified team members	Full
14	Gauld [31]	Advisory group/consultation	Indigenous/decolonising methods	Identified research clinician /research assistant		Community led or participatory research	Full
15	Hill [32]			Identified research clinician /research assistant Indigenous developed research tools	Authorship, feedback or editing	Identified team members	Full
16	Katzenellenbogen [33]		Indigenous specific ethics committee		Authorship, feedback or editing Affiliation	Identified team members	Full
17	Keel [34]				Affiliation	Identified team members	Full
18	Lee [35]	Advisory group/consultation	Indigenous specific ethics committee		Authorship, feedback or editing		Some
19	LoGiudice [36]		Indigenous specific ethics committee	Identified research clinician /research assistant	Affiliation		Some
20	Lukaszyk [37]	Advisory group/consultation	Indigenous specific ethics committee		Authorship, feedback or editing	Identified team members	Full
21	Lukaszyk [38]	Advisory group/consultation	Indigenous/decolonising methods Indigenous specific ethics committee	Identified research clinician /research assistant Indigenous developed research tools	Authorship, feedback or editing	Identified team members	Full
22	Lukaszyk [39]	Advisory group/consultation	Indigenous/decolonising methods Indigenous specific ethics committee	Identified research clinician /research assistant	Authorship, feedback or editing Affiliation	Identified team members	Full
23	Lukaszyk [40]		Indigenous specific ethics committee		Authorship, feedback or editing Affiliation		Some
24	Lukaszyk [41]	Advisory group/consultation	Indigenous specific ethics committee	Identified research clinician /research assistant	Authorship, feedback or editing	Identified team members	Full
25	McAuley [42]		Indigenous specific ethics committee		Authorship, feedback or editing Affiliation	Identified team members	Full
26	Moller [43]	Advisory group/consultation			Authorship, feedback or editing		Some
27	Moller [44]	Advisory group/consultation			Authorship, feedback or editing	Identified team members	Full
28	Moller [45]	Advisory group/consultation			Authorship, feedback or editing		Some
29	Moller [46]	Advisory group/consultation			Authorship, feedback or editing		Some
30	Phillips [47]	Advisory group/consultation	Indigenous/decolonising methods Indigenous specific ethics committee				Some
31	Ryder [48]		Indigenous/decolonising methods Indigenous specific ethics committee		Authorship, feedback or editing Affiliation	Identified team members	Full
32	Ryder [49]		Indigenous/decolonising methods Indigenous specific ethics committee	Identified research clinician /research assistant	Authorship, feedback or editing Affiliation	Identified team members	Full
33	Schultz [50]		Indigenous/decolonising methods Indigenous specific ethics committee	Identified research clinician /research assistant	Authorship, feedback or editing	Community led or participatory research Identified team members	Full
34	Shepherd [51]	Advisory group/consultation	Indigenous specific ethics committee				Some
35	Smith [3]		Indigenous/decolonising methods				Minimal
36	Thurber [52]		Indigenous specific ethics committee		Authorship, feedback or editing Affiliation	Identified team members	Full
37	Veli-Gold [53]		Indigenous/decolonising methods	Identified research clinician /research assistant	Authorship, feedback or editing	Identified team members	Full
38	Wallis [54]				Authorship, feedback or editing		Minimal
39	Williams [55]	Advisory group/consultation	Indigenous/decolonising methods	Identified research clinician /research assistant	Authorship, feedback or editing Affiliation	Identified team members	Full

**Fig. 2:**
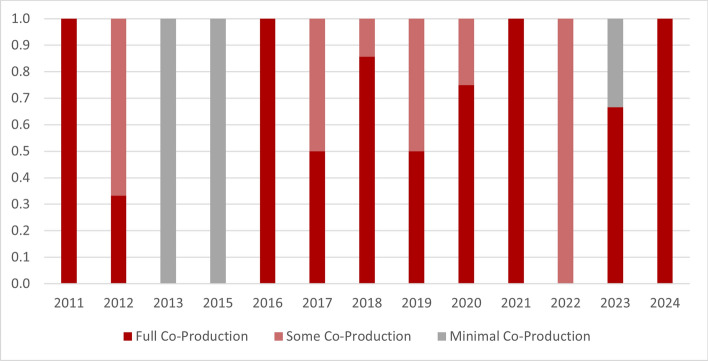
Proportion of co-production level over time. Source: developed by authors

Twenty-seven studies were considered to be fully co-produced as they either utilised co-production elements during each phase of research, or they had elements that covered the entire research process [18–26, 28, 30–34, 37–39, 41, 42, 48–50, 52, 53, 55]. The use of co-production elements across the whole research process is appearing to increase as the proportion of studies that are considered fully co-produced is rising (see Fig. 2).

Co-production in research is not a new or recent concept as elements are spread somewhat evenly across the publication dates. However, 67% of studies utilising Indigenous or decolonising methodologies were published after 2020, suggesting that these methods are becoming more widely accepted and used [3, 23, 24, 27, 28, 30, 44, 47–49, 53, 55].

Including an Aboriginal or Torres Strait Islander persons as an author or editor was the most frequently used element (31) [18–26, 28, 30, 32, 33, 35, 37–46, 48–50, 52–55], with 24 of those including the Aboriginal or Torres Strait Islander authors as members of the research team [18, 19, 21–26, 28, 30, 32, 33, 37–39, 41, 42, 44, 48–50, 52, 53, 55]. As a result, the analysis and dissemination (85%) [18–28, 30, 32–46, 48–50, 52–55], and the design and planning (79%) [3, 18–20, 23, 24, 26–28, 30, 31, 33, 36–53, 55] phases were the most likely to include co-production elements (see Table 8). During data extraction, there was another method that arose in the selected studies: engaging an Indigenous specific ethics committee, used by 27 studies [18, 19, 22–24, 26, 28, 30, 33, 35–52]. Of these studies, 59% were collecting new data [18, 19, 22–24, 26, 28, 30, 36–39, 41, 42, 47, 50, 51].

**Table 8:** Co-production elements grouped by their research phase

Research phase (co-production element)	Total	% of Total
Conceptualisation	13	33
*Advisory group or community consultation*	13	33
Design and planning	31	79
*Indigenous specific ethics committee*	25	64
*Indigenous or decolonising methods*	18	46
Empirical	19	49
*Aboriginal or Torres Strait Islander research clinician or research assistant*	17	44
*Aboriginal and Torres Strait Islander developed research tools*	5	13
Analytics and Dissemination	33	85
*Authorship, feedback or editing*	31	79
*Organisation affiliation*	16	41
Across all phases	26	67
*Community led or participatory research*	3	8
*Aboriginal or Torres Strait Islander team members*	25	64

Source: developed by authors

**Fig. 3:**
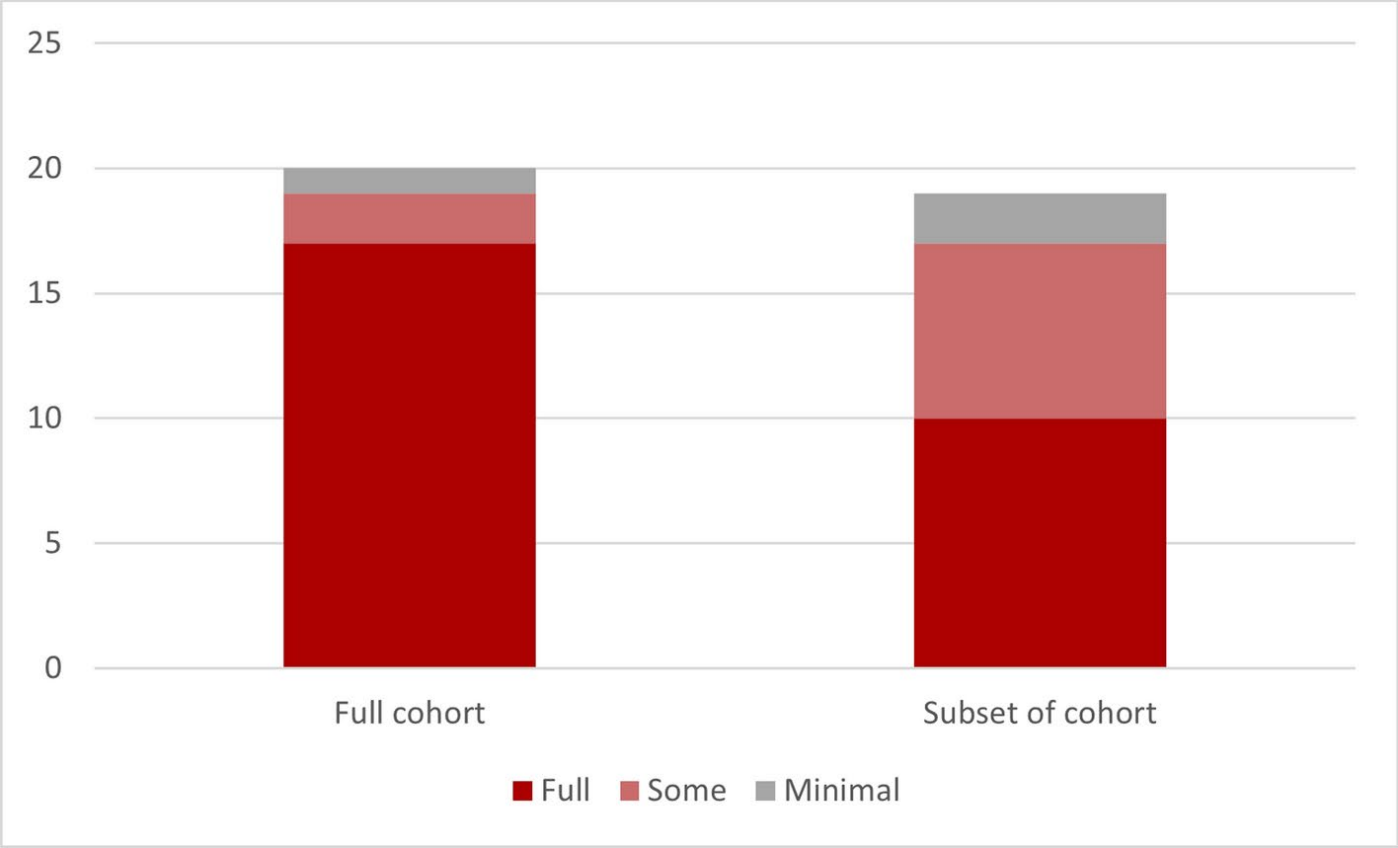
Target population by the amount of co-production. Source: developed by authors

Studies that targeted Aboriginal and Torres Strait Islander communities only (full cohort), were more likely to be fully co-produced (17 studies) [18–24, 28, 30–32, 37–39, 41, 52, 55] as shown in Fig. 3. Studies that incorporated Aboriginal and Torres Strait Islander peoples as a subgroup tended to have less co-production in the conceptualisation and empirical phases of the research.

**Fig. 4:**
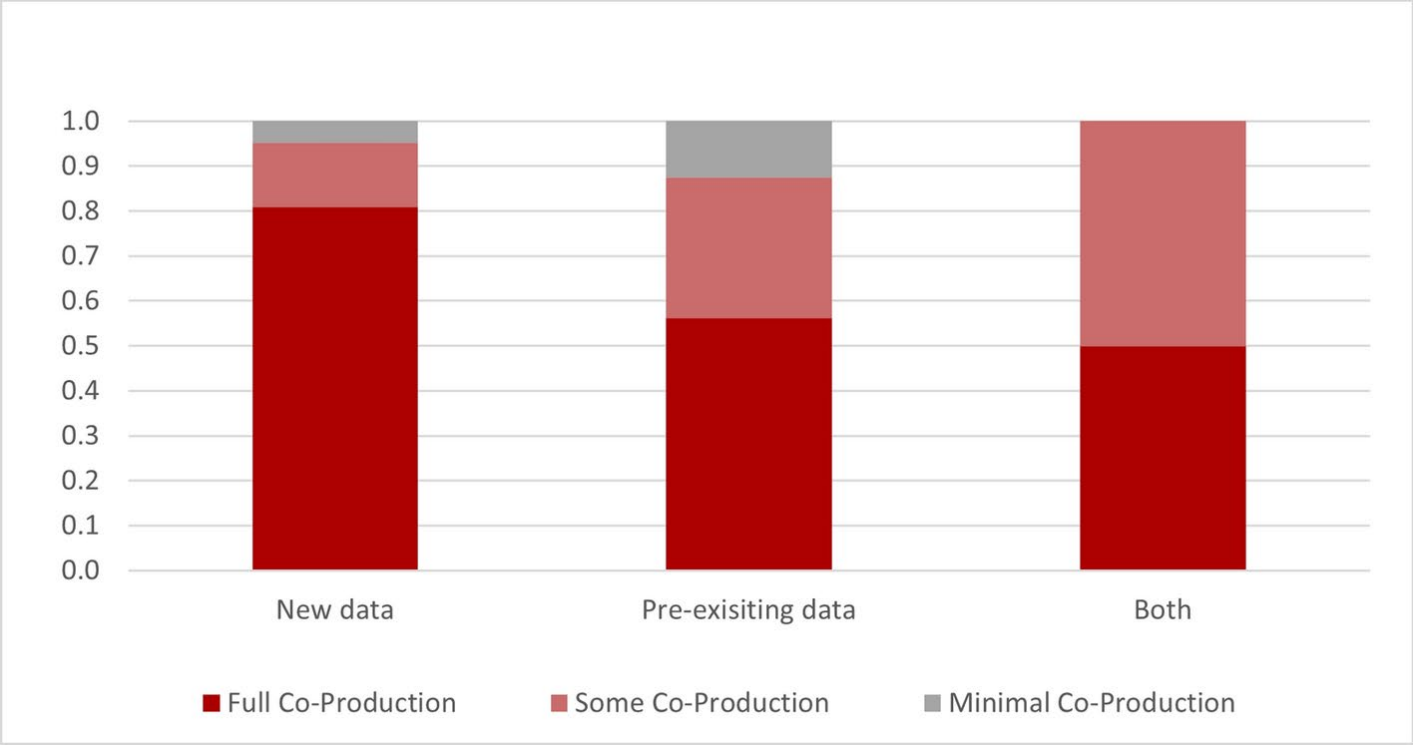
Proportion of data type and amount of co-production. Source: developed by authors

Additionally, studies that accessed pre-existing datasets were more likely to have less total co-production, than those collecting new data (see Fig. 4). However, there was one study that despite collecting new data, had minimal co-production throughout their research [3].

### Critical appraisal

Five studies contained high levels of Aboriginal and Torres Strait Islander perspective according to Harfield [17], with the highest score being 10.5 out of 14 (75%) [28]. All these papers were fully co-produced with community.

Unfortunately, there were 18 studies which captured none to low levels of Aboriginal and Torres Strait Islander perspective [3, 18, 25, 29, 32–36, 40, 42–46, 48, 51, 54], with one paper scoring zero [40]. Three of these were only minimally co-produced [40]. Interestingly, eight studies which were considered fully co-produced contained none to low levels of Aboriginal and Torres Strait Islander perspectives [18, 25, 32–34, 42, 43, 48], refer to Table 9.

**Table 9:** Critical appraisal of Aboriginal and/or Torres Strait Islander perspective within studies according to Harfield et al.’s [17] tool

Number	First author	Score percentage	Level of Aboriginal and Torres Strait Islander perspective capture
1	Angell [18]	32	None—low
2	Armstrong [19]	54	Medium
3	Bohanna [20]	36	Medium
4	Cheok [21]	39	Medium
5	Clapham [22]	68	High
6	Cochrane [23]	46	Medium
7	Coombes [24]	61	Medium
8	Cotter [25]	18	None—low
9	Dossetor [26]	46	Medium
10	Edmonston [27]	43	Medium
11	Esgin [28]	75	High
12	Falster [29]	7	None—low
13	Fraser [30]	57	Medium
14	Gauld [31]	71	High
15	Hill [32]	29	None—low
16	Katzenellenbogen [33]	7	None—low
17	Keel [34]	14	None—low
18	Lee [35]	18	None—low
19	LoGiudice [36]	21	None—low
20	Lukaszyk [37]	43	Medium
21	Lukaszyk [38]	54	Medium
22	Lukaszyk [39]	71	High
23	Lukaszyk [40]	0	None—low
24	Lukaszyk [41]	43	Medium
25	McAuley [42]	7	None—low
26	Moller [43]	14	None—low
27	Moller [44]	18	None—low
28	Moller [45]	7	None—low
29	Moller [46]	11	None—low
30	Phillips [47]	39	Medium
31	Ryder [48]	32	None—low
32	Ryder [49]	46	Medium
33	Schultz [50]	57	Medium
34	Shepherd [51]	14	None—low
35	Smith [3]	25	None—low
36	Thurber [52]	54	Medium
37	Veli-Gold [53]	57	Medium
38	Wallis [54]	11	None—low
39	Williams [55]	71	High

There were two questions in Harfield et al.’s [17] tool which none of the studies properly addressed: question 6, *did the researchers negotiate agreements in regards to rights of access to Aboriginal and Torres Strait Islander people’s existing intellectual and cultural property,* and question 7, *did the researchers negotiate to protect Aboriginal and Torres Strait Islander people’s ownership of intellectual and cultural property created through the research?* The majority of papers were marked as unclear about the ownership of information and did not discuss in detail the sovereignty, protection or governance of collected data. This does not mean that it did not occur, however, it has resulted in lower scores for the studies.

## Discussion

This is the first scoping review consolidating the available evidence on the use of co-production methodologies in research within the unintentional injuries space for Australian Aboriginal and Torres Strait Islander communities. Over time co-production elements are relatively evenly distributed across the injury studies. However, there was a clear spike in publications in 2018 and 2021. This may be attributed to the 10-year review of the Closing the Gap strategy [4] and the recommendations for policies provided by community in 2020 [56, 57]. Research around these times may have been part of the information and evidence gathering to check in on the “progress” of the policies.

Despite all studies having Aboriginal and Torres Strait Islander engagement and co-production within their methodologies, not all research topics were informed or led by community needs. Inclusion of Indigenous standpoint more in the conceptualisation of research may change how injury and (physical) trauma are viewed, altering which conditions and interventions are investigated. Only one selected study in this review was initiated by the community; the researchers were asked to review emergency department presentations rather than a specific injury or issue [26]. This evidence suggests that while unintentional injury research is co-produced, culturally safe and helpful, investigations may not be targeting topics that are of community interest.

However, the incorporation of more individualistic measures such as prevention, rehabilitation and community-based services, means there is greater recognition of the different needs between communities [30]. Moreover, the introduction of Aboriginal and Torres Strait Islander services such as Aboriginal Medical Services, Aboriginal Community Controlled Health Organisations, Indigenous Liaisons, nurse navigators and coordinators has provided the ability to act on these differing needs and create culturally safe spaces within healthcare.

For example, falls has been discussed in literature as a major concern voiced by Aboriginal and Torres Strait Islander communities [41], so there is great emphasis on developing and maintaining effective falls prevention programs that are supportive of and by the community [18, 32, 39, 41]. Falls can lead to significant injury and long-term disability such as brain injuries causing strain on families and communities [20]. The Ironbark program was developed to address the lack of culturally safe fall-prevention services in Aboriginal and Torres Strait Islander communities and undergoes evaluation with users to ensure its ongoing relevancy and usefulness [58].

Additionally, burn injuries are beginning to be considered from a cultural standpoint as fire is an important cultural component for many Aboriginal and Torres Strait Islander communities [55]. Traditional medical and health research is based on Western models, which create ‘gaps’ when communities do meet the standard that is not based on their needs [59]. Ryder et al. [48, 49] has been incorporating weaved research methodologies to understand burns within Aboriginal and Torres Strait Islander children to implement relevant, culturally appropriate and effective solutions.

### Co-production and perspective

While Aboriginal and Torres Strait Islander persons on the writing team or research team were the most commonly used co-production elements, the usage of this method has remained consistent over time. The process of implementing co-production elements in research focusing on Aboriginal and Torres Strait Islander people can be challenging and time-consuming because many communities are hesitant to participate due to historical issues of exploitation and colonisation [6, 60]. To address this issue/problem, including Aboriginal or Torres Strait Islander people on the writing or research team may be the most efficient, safest, and widely accessible option in the current research landscape. In doing so, it centres and privileges Indigenous Knowledges within the research methodology, better incorporating Ways of Knowing, Doing and Being through culturally grounded approaches [60]. Additionally, it ensures strengths-based approaches in the research, ultimately helping to decolonise research process and literature [1].

Decolonisation and the process of decolonising literature is not well understood within Western Knowledge systems [1]. One of the key messages within decolonising literature is valuing and empowering Aboriginal and Torres Strait Islander voices in the literature. Co-production is a central part of this process and is needed to drive culturally aware policy change [4]. In 2017, the Uluru Statement from the Heart was released describing ongoing issues with oppression and calling for structural changes and recognition to allow for self-governance [61]. Western research methodology is not an appropriate way of understanding Aboriginal and Torres Strait Islander communities [59]. Instead, Indigenous Knowledge Systems and Methodologies need to be incorporated and valued within research, academia, and policy to change deficit narratives and empower communities through culturally safe processes [59, 62]. To achieve this, the best approach is to integrate full co-production requirements to include all voices in the discussion to promote truth-telling and knowledge sharing [2, 62].

Clapham, Bennett-Brook [22] provided an example of this in their methodology, ensuring that there was space and time for capacity building between Aboriginal Family Workers and the research team. Relationship building and knowledge sharing was central to the methodology of the research as it aided in decolonising the research process. Incorporating this component of the methods within the published work is also significant as it highlights approaches that are not usually discussed in literature.

Acknowledging that co-production can be challenging, and that the burden of decolonisation should not lie solely with Aboriginal and Torres Strait Islander peoples, Western researchers have a responsibility to treat Indigenous data ethically and responsibly [9]. Therefore, where full co-production is not possible, Western researchers should endeavour to use or incorporate Indigenous or decolonising methods wherever data about Aboriginal and Torres Strait Islander peoples are being used. The use of Indigenous and decolonising methods, and Indigenous developed research tools are on the rise within research with Storytelling, Yarning and Dadirri often used to help decolonise the data collection and ensure cultural safety of the participants. Phillips et al. [47] describes how different interviewing styles were used between communities, recognising the different needs of their participants. Additionally, Thurber et al. [52], specifically utilised culturally safe pre-existing data in their analysis, understanding that not all data is collected the same and there is inherent bias in methodology. However, as evidenced by Edmonston et al. [27], Phillips et al. [47] and Smith et al. [3], Indigenous and decolonising methods can be used without having Aboriginal or Torres Strait Islander persons in the writing or research team, reducing the co-production value. Despite the grounded approach and privileging of Indigenous voice, it means this element cannot be taken alone as a sign of full co-production.

### The role of data

National and statewide datasets, such as the Australian Bureau of Statistics or public hospital registries, are the most frequently accessed when using pre-existing data [25, 26, 29, 33, 35, 40, 43–46, 48, 49, 53, 54]. However, the underlying methodology and bias within these collections are not always considered. Basing research solely on these kinds of collections can result in a skew in information as data may not be culturally safe or accurate [63]. There is a reluctance to self-identify in these datasets as there is a lack of transparency and impact historical legacies are still felt within community [64]. The smaller sample size can mean anomalies are highlighted. Additionally, common research techniques within Western Methodologies can produce misinterpretations and misinformation [63]. An example of this is the aggregation and homogenisation of Aboriginal and Torres Strait Islander peoples as there are many different communities each with different customs, traditions and cultures [65]. Hence grouping all communities removes contextual factors and perpetuates the idea of likeness among all peoples [63, 65, 66]. Othering stems from this aggregation and homogenisation as it compares communities to the ‘established white norms’ or population model [62].

Moreover, when using pre-existing data, there were less co-production elements utilised in the research, with no community led studies in this review. There is a belief that cultural competency is not necessary or that culturally safe research only needs to occur when new data is collected, rather than at all times. This is further evidenced by the number of studies that utilise new data collection and engaged an appropriate advisory group [20, 22, 27, 31, 37–39, 41, 47, 55] or included identified persons in the research team [18, 19, 22–24, 28, 30, 32, 37–39, 41, 42, 50, 55] compared to those that accessed pre-existing data (only two consulted with advisory groups [29, 35], and nine included identified persons in the research team [21, 25, 26, 33, 43, 48, 49, 52, 53]). Bias within data collection often goes unacknowledged without reduction measures to improve quality or accuracy.

Indigenous Data Sovereignty targets these issues at the core, as it is about the ownership, protection and dissemination of Aboriginal and Torres Strait Islander data and information [6, 67]. Recently, there has been work to put safeguards in place to protect Aboriginal and Torres Strait Islander data, so it can be safely accessed alongside mainstream information [68]. Historically, this lack of protection has resulted in the exploitation of Aboriginal and Torres Strait Islander information and data as narratives of deficit and disadvantage are perpetuated [6, 59]. The vast amount of research and investigation conducted on communities continue issues of racism through literature and contribute to the distrust in Western research [1, 67]. Much of this distrust has stemmed from Western researchers pushing agendas on communities and using research as a justification for oppression rather than seeking true solutions [59, 66].

Pre-existing data is a useful tool which can save resources and provide information for retrospective research. Appropriate measures need to be taken to reduce bias and support culturally safe research when accessing data [63]. Co-production methods such as advisory groups, engagement with Indigenous specific ethics committees, inclusion of identified persons on the research team and supporting community led research will help to minimise the bias as information can be better analysed and power is re-balanced [10, 11, 13]. Incorporating the necessity to co-produce or include co-production methods with all research pertaining to Aboriginal and/or Torres Strait Islander communities into research and governance policies will empower Indigenous Data Sovereignty. The Australian Government have begun formulating such policies to encourage co-production and partnerships [69].

The development of analytic tools for the purposes of analysing and interpreting Aboriginal and Torres Strait Islander data specifically appears to be a gap in the literature. Similar to Harfield, Pearson [17] utilising a tool to highlight bias within datasets and potentially reduce that bias would be beneficial for all research. While much of the inaccuracies are known (e.g., low rates of self-identification), the ability to quantify the impact of the bias on potential outcomes or conclusions of research can highlight the limitations and usefulness of the dataset (and collection methods).

Despite the necessity to research with Aboriginal and Torres Strait Islander peoples, not on communities, many articles were excluded during the screening process due to the lack of co-production. Within the process of this review, there were eight unintentional injury studies that included information about Aboriginal and Torres Strait Islander peoples that were excluded due to the lack of co-production in their methodology. The absence of co-production presents a problem for the interpretation and explanation of data as literature in Western Systems tends to have a deficit frame [60]. Injury trends are often discussed without providing context such as history, and communities are often aggregated and homogenised to remove cultural differences [63, 65]. The meaning of the data can be lost resulting in incorrect or inaccurate conclusions.

However, even with perceived full co-production within the research methodology, Aboriginal and Torres Strait Islander perspectives are still not always accurately reflected in disseminated information. As assessed using a recognised critical appraisal tool [17], it was found that even among the studies which had full co-production, Aboriginal and Torres Strait Islander perspectives were only moderately captured and expressed. There was the obvious trend; the less co-production within a study, the less perspective was captured and, it is worth noting it is not possible to capture and express Aboriginal and Torres Strait Islander perspectives without co-production.

There are inherent problems within Western Methodologies that inhibit important discussions with Indigenous Methodologies [62]. Information and data governance, sovereignty and custodianship are not often discussed within Western-based literature but are of great significance within Aboriginal and Torres Strait Islander research. The history of exclusion and lack of transparency of research methods has meant communities are unable to access information that is about their peoples [70]. Unfortunately, this is an area that still requires much work, as none of the selected studies really addressed these topics within their methods.

### Strengths and limitations

This scoping review had several strengths. This review was registered with the Open Science Framework to ensure transparency in the methods. Secondly, the PRISMA-ScR framework was followed to ensure accuracy of the undertaken steps. The search strategy was systematic, comprehensive, and included peer-reviewed articles. The inclusion of a co-author and team member with Aboriginal and Torres Strait Islander background is a significant factor in the success of this scoping review, who provided valuable guidance, insight and efforts throughout the study. The research was culturally grounded and respected Aboriginal and Torres Strait Islander perspectives thanks to the collaboration, which enhanced its overall quality and relevance. Finally, the findings are important for highlighting the gaps and potential areas for further work in co-production of injury research. This will provide better guidance for future research in this field.

A limitation of this study is that elements of co-production were only included if they were explicitly stated in the article. Therefore, we could have missed some studies that did include elements of co-production. However, where co-production was used it was usually clearly described in the methods, contributions and or acknowledgments sections. We found that most studies which included substantial involvement of Aboriginal and Torres Strait Islander community members described it in some detail. It is more likely we could have missed whether some researchers and authors were Aboriginal or Torres Strait Islander people, as this is less often explicitly stated. Therefore, we are unlikely to have misclassified studies that did use substantial elements of co-production.

This review includes studies related to unintentional injuries only, and so may not be generalisable to other health areas. However, it is likely that there are similarly low levels of inclusion of Aboriginal and Torres Strait Islander community members in the design of research and interpretation of data related to other health issues. While we excluded studies about intentional injuries, as it is a sensitive area, we note that poisoning can sometimes be intentional or unintentional and the study on poisoning may have included data on intentional poisonings.

We acknowledge that most of the researchers on this project are not Aboriginal or Torres Strait Islander people, and so this may also have biased our interpretation of the data in this review. Applying our frameworks to this review paper, our research would be considered fully co-produced due to the research and writing team incorporating an Aboriginal or Torres Strait Islander person and utilising an Indigenous developed analysis tool. However, according to Harfield et al. [17], we have only captured a moderate level of Aboriginal and Torres Strait Islander perspective (36%).

### Recommendations and future steps

There is a clear gap in studies that utilise pre-existing datasets and co-production within injury research. Despite current progress, Aboriginal and Torres Strait Islander information is not protected enough to ensure data is not used in harmful ways. As Indigenous Data Sovereignty and Governance structures are implemented, barriers to access data will arise to enforce some engagement [68]. However, due to bias within datasets, using co-production elements such as Aboriginal and Torres Strait Islander research assistants, analysts and team members, alongside Indigenous developed analysis tools will centre Indigenous Knowledges and voices within the research. It will enable a comparison with the lived experience to draw strength from Indigenous standpoints [71]. Moreover, these barriers will facilitate discussion about data custody to address questions about ownership and transparency of process as outlined in the Harfield et al.’s tool [17].

The next step of this research is to assess the effectiveness of co-production elements within injury research settings, in terms of investigation rigour and meaningfulness of the research. It is important to understand how different co-production methods improve the meaningfulness and cultural appropriateness of the research [10]. This will better inform researchers of the best practices in co-production methods specifically with Aboriginal and Torres Strait Islander peoples, and ensure teams are using the appropriate methods for their investigations.

## Conclusion

This review provides a valuable comprehensive overview of the current state of co-production in unintentional injury research that involves Aboriginal and Torres Strait Islander communities. Incorporating Indigenous Knowledges and supporting decolonisation of research can be achieved by including Indigenous individuals in the research teams, despite the challenges of historical exploitation. While some engagement with community or use of Indigenous methods helps to ground Indigenous voice within the research process, it is not currently enough to address the lack of meaningful research, the needs of community or capture Aboriginal and Torres Strait Islander perspectives. The diversity of co-production elements is improving over time with the recent development of Indigenous specific tools and the work undertaken to implement Indigenous Data Sovereignty, facilitating better research outcomes from enhanced research methodologies. The inclusion of meaningful Indigenous perspectives should be prioritised in future research, with the methodologies and outcomes being culturally safe and relevant.

## Supplementary Information

Supplementary Material 1.

## Data Availability

All data generated or analysed during this study are included in this published article (and its supplementary information files).

## Abbreviations

CINAHL: Cumulated Index to Nursing and Allied Health Literature
MeSH: Medical Subject Headings
PRISMA-ScR: Preferred Reporting Items for Systematic reviews and Meta-Analyses extension for Scoping Reviews

## Declarations

### Disclaimer

Please note that due to the inherent nature of the methodology in literature reviews, this paper has/may include deficit-framing language.
